# Ubidecarenone-Loaded Nanostructured Lipid Carrier (UB-NLC): Percutaneous Penetration and Protective Effects Against Hydrogen Peroxide-Induced Oxidative Stress on HaCaT Cells

**DOI:** 10.3390/ijms19071865

**Published:** 2018-06-25

**Authors:** Jianmin Wang, Huiyun Wang, Qiang Xia

**Affiliations:** 1 School of Pharmacy, Jining Medical University, Rizhao 276826, China; wang__huiyun@126.com; 2 State Key Laboratory of Bioelectronics, Southeast University, Nanjing 210096, China; xiaq@seu.edu.cn

**Keywords:** ubidecarenone, nanostructured lipid carrier, antioxidation, reactive oxygen species, percutaneous penetration

## Abstract

The objective of the investigation was to evaluate the percutaneous penetration of a ubidecarenone-loaded nanostructured lipid carrier (UB-NLC) and to illuminate the protective effects of UB-NLC for amelioration of hydrogen peroxide-induced oxidative damage on HaCaT cells. Ubidecarenone (UB) was encapsulated in a nanostructured lipid carrier (NLC), which was manufactured by homogenization. The morphological and dimensional properties of the prepared UB-NLC were studied by freeze-fracture transmission electron microscopy (FF-TEM) and photon correlation spectroscopy (PCS). Percutaneous penetration of UB-NLC was carried out by the Franz diffusion cells method. The change of cellular morphology was identified through a non-invasive time-lapse imaging system. The assessment was achieved via the evaluation of the levels of oxidative stress markers: reactive oxygen species (ROS), superoxide dismutase (SOD), glutathione peroxidase (GSH-PX), and malondialdehyde (MDA). Percutaneous penetration of UB loaded in NLC formulation was enhanced in comparison to free UB. Preincubation of HaCaT cells with UB-NLC attenuated the level of intracellular generation of ROS. Lipid peroxidation was diminished by UB-NLC via inhibition of MDA formation. Pretreatment of cells with UB-NLC reestablished the activity of cellular antioxidant enzymes (SOD and GSH-PX). On the basis of the investigation conducted, results suggest that formulating UB as NLC is advantageous for topical delivery and treatment of oxidative stress-induced human diseases.

## 1. Introduction

Reactive oxygen species (ROS) are engendered in the oxidation-reduction procedure [1]. ROS, as products of the oxidation and peroxidation process, are instantaneously detoxified by ROS scavengers and antioxidants under physiological conditions [2]. For example, types of ROS, such as superoxide radical, hydrogen peroxide, hydroxyl radical, are generated in aerobic living organisms and are present in the cell under balance with oxidation and antioxidation procedure [3]. ROS are the by-products of cellular metabolism, which play a fundamental role in gene transcription and cell signaling, in addition to growth factor and hormone action, the production of cytokine, as well as immune-modulation, etc. [4].

Oxidative stress (OS) results from the incongruity of redox equilibrium between the manufacture of ROS and the capability of resistance against them [5,6]. ROS are the main free radical sources of oxidative stress [7]. OS is induced by an overproduction of ROS, which may bring about direct or indirect ROS-mediated oxidative impairment, for example, disruption of the cellular membrane, changes in nucleic acids, protein denaturation, and lipid peroxidation; it also has been implicated in cells apoptosis and physiological dysfunction [4,8,9,10,11,12].

A given mass of oxidative damage occurs under physiological conditions; however, the degree of oxidative damage increases throughout the aging procedure and in the company of some diseases as the effectiveness of antioxidation and restoration mechanisms decreases [3]. Lipid peroxidation is a conventional mechanism of cellular damage and serves as an indicator of oxidative stress [13]. Malondialdehyde (MDA) acts as a main oxidation product of peroxidized polyunsaturated fatty acids and serves as a biological indicator of oxidative stress [13]. The level of MDA can be regarded as the level of lipid peroxidation [9,13]. When MDA reacts with proteins and nucleic acids, it also brings about cell dysfunction [9]. Superoxide dismutase (SOD), the first line of protection against free radicals, has ROS-metabolizing activity and can catalyze the dismutation of superoxide radicals into hydrogen peroxide and molecular oxygen [6,9]. Glutathione peroxidase (GSH-PX) catalyzes the degradation of hydrogen peroxide and lipid peroxides [6]. GSH-PX transforms hydrogen peroxide into oxygen and water through converting reduced glutathione into oxidized glutathione [5,6]. GSH-PX can also defend against lipid peroxidation via a termination of the chain reaction and elimination of lipid peroxides from the cellular membrane [5].

An achievable approach to guard against ROS-induced cellular damage is to enlarge the oxidative protection capability via intake of antioxidants [6]. Several exogenous antioxidants have been researched in terms of their potential advantages to defend against oxidative injury [14]. The activity of enzymatic antioxidant systems, including SOD and GSH-PX, could modulate the balance of ROS with antioxidants [3].

Ubiquinone (UB), also known as coenzyme Q10 [15,16], is a coenzyme that is regarded as a vitamin-like lipid soluble substance [17,18,19]. UB is a vital cofactor in the mitochondrial respiratory chain and plays a fundamental role in oxidative phosphorylation to allow ATP production [1,20,21]. Accordingly, UB plays a critical role in energy biosynthesis within the cells and serves as a cofactor in cellular bioenergetics [18]. UB is a compound comprised of a redox-active quinoid moiety and a hydrophobic tail [13], which is a powerful lipophilic antioxidant [21]. It serves as a redox agent for protons and electrons by the electron transport chain [5]. UB not only plays an essential role in energy metabolism but also acts as an antioxidant that scavenges free radicals [9]. The antioxidation of UB originates from its scavenging of free radicals and serves as an essential quencher of ROS [6,13,21]. UB serves as an antioxidant capable of either directly reacting with radicals or recycling and regenerating tocopherol and ascorbate [5,18]. UB may restrain lipid peroxidation and oxidative stress damage through an adjustment of enzymatic activities (such as SOD and GSH-PX), averting the production of lipid peroxyl radical to defend against ROS-induced lipid peroxidation [5,9,17,21]. The important role of UB in cell membrane stabilization is its prevention of lipid peroxidation and maintenance of cellular function [9]. UB can restrain the oxidation of lipids, proteins, and DNA [5]. UB deficiency may bring about mitochondrial dysfunction, leading to the generation of ROS. The capability of ROS eliminating is weakened, potentially further aggravating the accumulation of ROS because of UB deficiency [1]. The fundamental roles of UB include energy biosynthesis and conversion, antioxidant regeneration, antioxidant activity, and its status as a cofactor for enzymatic reactions; accordingly, it has found applications as a drug, nutriment supplement, and cosmetic agent [2,18].

Nanostructured lipid carriers (NLC) are composed of lipids in the solid state at room temperature which have been blended with lipids in a liquid state [22,23]. The lipid ingredients serve as part of lipid nanoparticle formulation and have been subjected to numerous safety evaluations and are currently in use in FDA-approved products [22]. During the preparation of NLC, solid lipids are melted and crystallized; next, the diversely structured liquid lipids bring about the production of a highly defective and disordered lipid matrix structure which can offer considerable cavities to load poor soluble drugs [22,23]. NLC can served as a versatile drug delivery carrier, with drug-loading capacity, stability, high biocompatibility, and prolonged drug residence at the target location; it also features minimal drug expulsion during storage [24].

One drawback to UB is its poor topical bioavailability, which is attributable to its large molecular weight and high hydrophobic nature. Nevertheless, several previous attempts have been made to ameliorate topical bioavailability as well as patient compliance in the employment of NLC formation [18,25,26]. The investigation assessed the supposition whether or not UB-NLC administration diminished oxidative stress on HaCaT cells. The present study was designed and performed to estimate the probable therapeutic role of UB-NLC formulation against ROS-mediated oxidative stress. Several aspects of UN-NLC activity were studied including its antioxidation in terms of levels of ROS, SOD, GSH-PX, MDA, etc. As part of our investigation to illuminate the antioxidation potential of produced UB-NLC, we studied whether it could protect against hydrogen peroxide-induced oxidative stress on HaCaT cells.

## 2. Results and Discussion

### 2.1. Microstructure Observations and Size Characterization

**Figure 1:**
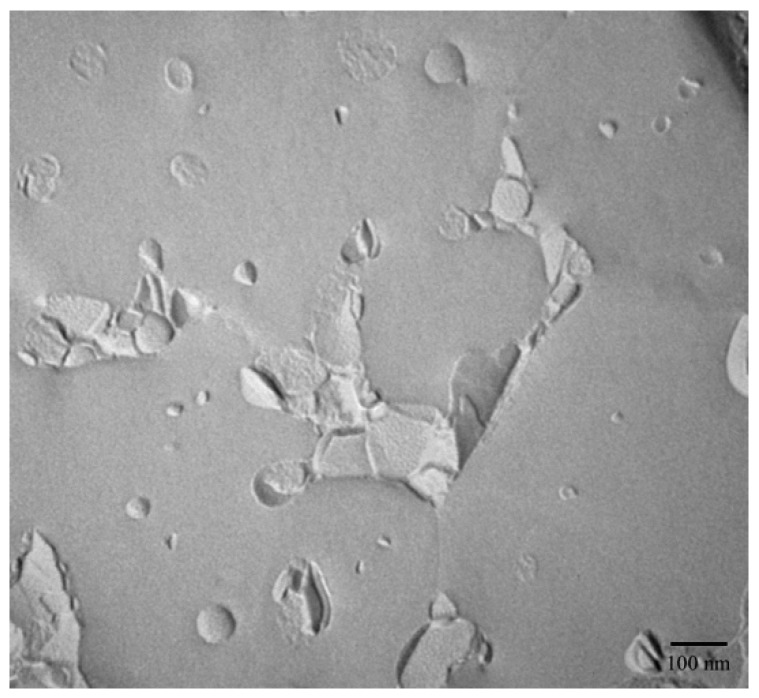
Typical freeze-fracture transmission electron microscopy (FF-TEM) micrographs of ubidecarenone-loaded nanostructured lipid carrier (UB-NLC).

**Figure 2:**
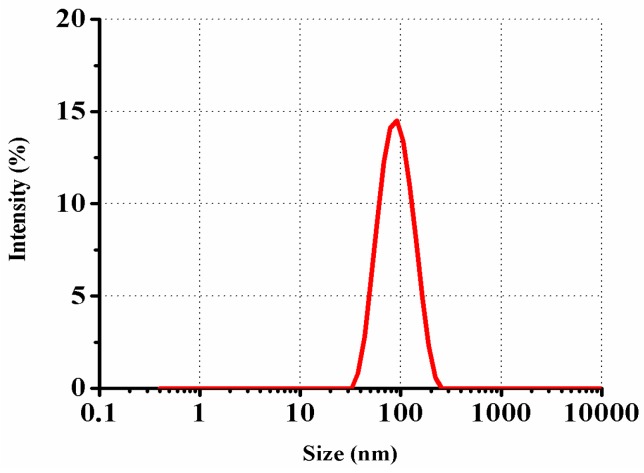
Size distribution representative profile of intensity from UB-NLC measured using photon correlation spectroscopy (PCS).

The morphology and size of NLC were characterized using FF-TEM and PCS, respectively. The FF-TEM determination provides an unrivaled possibility to visualize the morphology of UB-NLC. As shown in Figure 1, for typical FF-TEM micrographs of UB-NLC with samples freeze-fractured, spherical-like structures can be observed, indicating that the UB-NLC formation had been developed. Using PCS for size determination, the results presented the size of nanoparticles, as seen in Figure 2. These results corresponded well with those acquired from the FF-TEM observation, with the results exhibited in Figure 1.

### 2.2. Percutaneous Penetration

**Figure 3:**
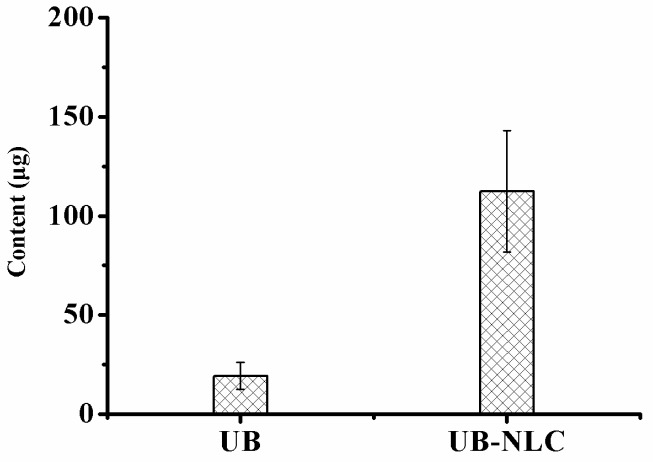
Skin deposition of UB in NLC compared with ethanol solution in dermatomed rabbit skin. Data have been expressed as the mean and standard deviation.

**Figure 4:**
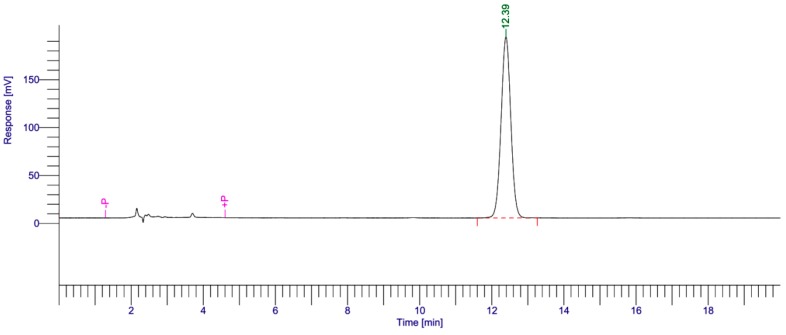

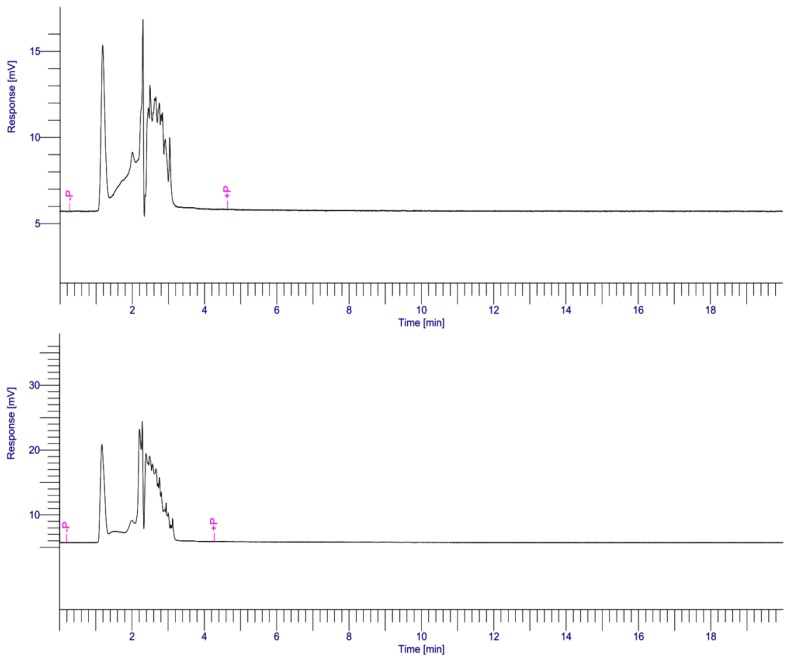
Representative chromatogram profiles of free UB solution (**upper**), receptor medium of free UB group (**middle**) and UB-NLC group (**lower**) studied under optimum conditions. The peak eluted at about 12.4 min, which was the characteristic peak.

The obtained skin deposition parameters of UB from the optimal NLC formation compared with UB solution are collectively represented in Figure 3. The results illustrate that the skin served as a reservoir for the drug (UB). As represented in Figure 3, UB revealed higher skin deposition from the studied NLC in comparison to the UB solution. The skin deposition quantity of UB was 112.5 μg from NLC versus the UB solution, which was equivalent to 19.3 μg. Results indicate that UB-NLC considerably augmented the skin deposition of the drug by nearly 5.8-fold more than that of UB solution. After exposure, the mean concentration of UB in the receiver compartment was under the level of detection (LOD) for both UB and UB-NLC formulation (Figure 4). The data could be ascribed to the elastic and flexible characteristic of UB-NLC, which enabled it to pass through the skin pores to increase the penetration of UB [27]. In addition, NLC can serve as a penetration enhancer, in which NLC punches through the stratum corneum and, afterwards, adjusts the fluidity of the stratum corneum lipids. NLC also alters the intercellular lipid lamellae, enabling penetration of the drug into and through the stratum corneum [28,29]. Nanoparticles (NLC) also can enter the stratum corneum, allowing the core materials (such as the drug) to be delivered into the stratum corneum; the water phase of nanoparticles could hydrate the skin to facilitate drug penetration [28,29,30].

Some mechanisms can elucidate the detected augmentation in penetration delivery of UB from NLC. Drug permeation into the skin is influenced by the physicochemical characteristics of nanoparticles, for example, size, morphology, surface potential, solubility, and formation of the drug delivery system [29,30]. Both the size and charge of nanoparticles are significant factors for uptake of skin absorption. Particle size is the most crucial characteristic which affects skin permeation [30]. The nano-sized particles may serve as drug delivery carriers whereby intact nanoparticles adsorb and adequately blend with the skin, transferring the nanoparticle-loaded drug into the skin [28]. The smaller size of nanoparticles offers better percutaneous penetration in a bigger surface area [28,30,31]. Nanoparticles penetrate the skin through a variety of routes: across the corneocytes (intracellular/transcellular route), between the corneocytes (intercellular route), and by the appendages (such as sweat glands or hair follicles) [28]. Furthermore, hair follicles are regarded as a predominant shunt route for the penetration of nanoparticles into the skin [30].

### 2.3. Antioxidant Activity of UB-NLC against Oxidative Damage

UB-NLC protects against oxidative stress-induced cellular oxidative damage. This investigation determined the additional protective effect of UB-NLC on cellular oxidative damage. We measured whether UB-NLC demonstrated an enlarged, or at least a similar, protective activity against oxidative stress-mediated cell damage. To determine the oxidative stress-mediated cell damage, HaCaT cells were pretreated with hydrogen peroxide. Time-lapse imaging assays were implemented on HaCaT cells to detect the cellular reply to different samples. The method allowed for real-time and non-disturbance in situ time-lapse determinations for the observance of morphological alterations during UB-NLC protection against oxidative damage. The process was employed iteratively over all scheduled time points, segmenting the time lapse pictures and following the cells.

**Figure 5:**
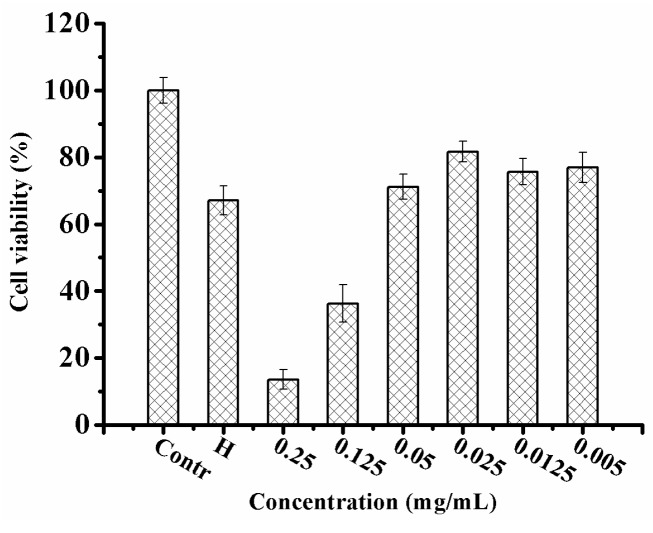
Protective effect of UB-NLC against hydrogen peroxide-induced oxidative stress on HaCaT cells.

**Figure 6:**
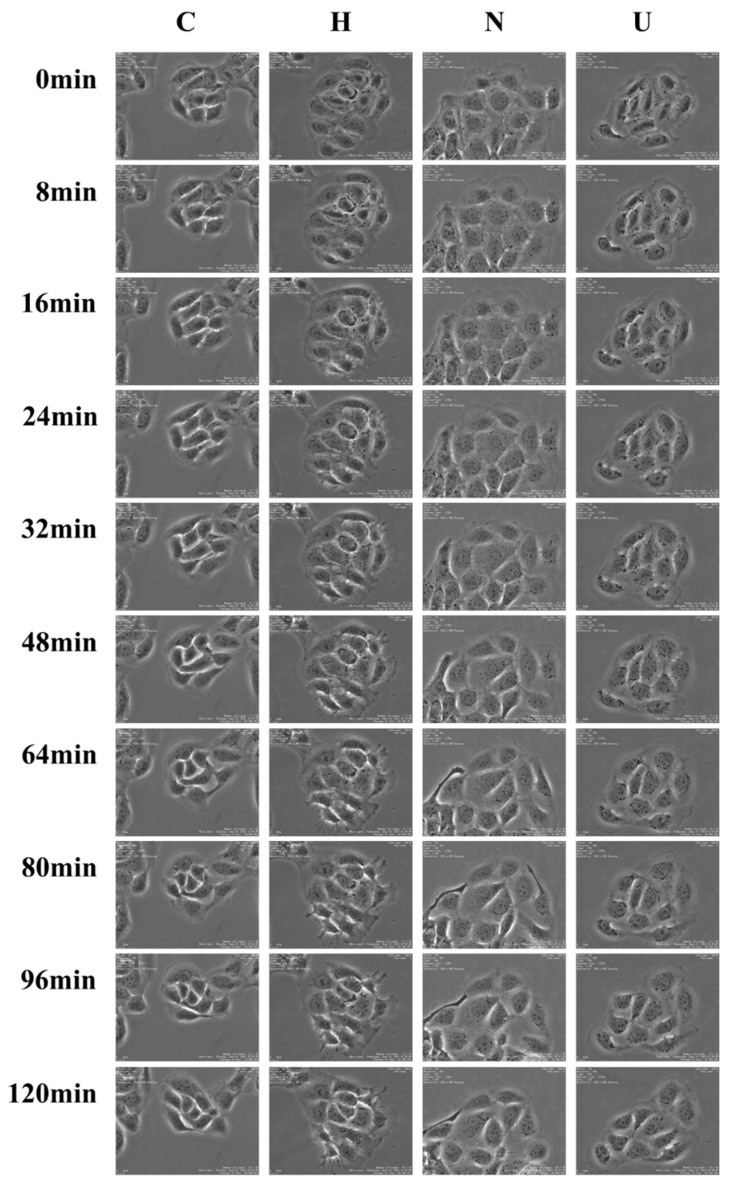
Representative photographs (objective 40×) from time-lapse imaging analysis of UB-NLC protecting against oxidative damage. C: Normal culture group; H: Only oxidative damage group; N: UB-NLC (0.025 mg/mL)-treated group; U: Free UB-treated group.

Treatment with UB-NLC revealed protective activity against hydrogen peroxide-mediated oxidative injury in HaCaT cells when evaluated via a cell viability measurement (Figure 5) and time-lapse experiment (Figure 6). UB-loaded nanostructured lipid carriers offered a protective effect, augmenting cell viability. The findings indicate a correlation amongst ROS-scavenging activity, free radical-quenching effect, and the increased resistance to hydrogen peroxide-mediated oxidative impairment [6]. HaCaT cells viability experiments and the sustained dynamic image assessment of the cells revealed a high biocompatibility and capability of ameliorating the protective activity of UB-NLC toward oxidative damage. Antioxidation ability may play a major role for UB-NLC in contributing to the assuagement of cellular oxidative stress. However, because the administration of several exogenous substances cannot replace entirely natural antioxidant protection systems, the complex antioxidant system is imminently demanded [2].

### 2.4. Monitoring the Level of Reactive Oxygen Species in HaCaT Cells

Intracellular ROS accumulation was assessed by 2′,7′-dichlorofluorescein diacetate (DCFH-DA) fluorescent dye [32]. DCFH-DA can freely pass through the cellular membrane and is enzymatically hydrolyzed via intracellular esterases to convert into non-fluorescent DCFH [33]. ROS can oxidize DCFH to the highly fluorescent compound, 2′-7′-dichlorofluorescein (DCF) [34], whose intensity of fluorescence is directly proportionate to the amount of intracellular ROS, such as hydroxyl radical, hydrogen peroxide, or peroxynitrite [35,36].

**Figure 7:**
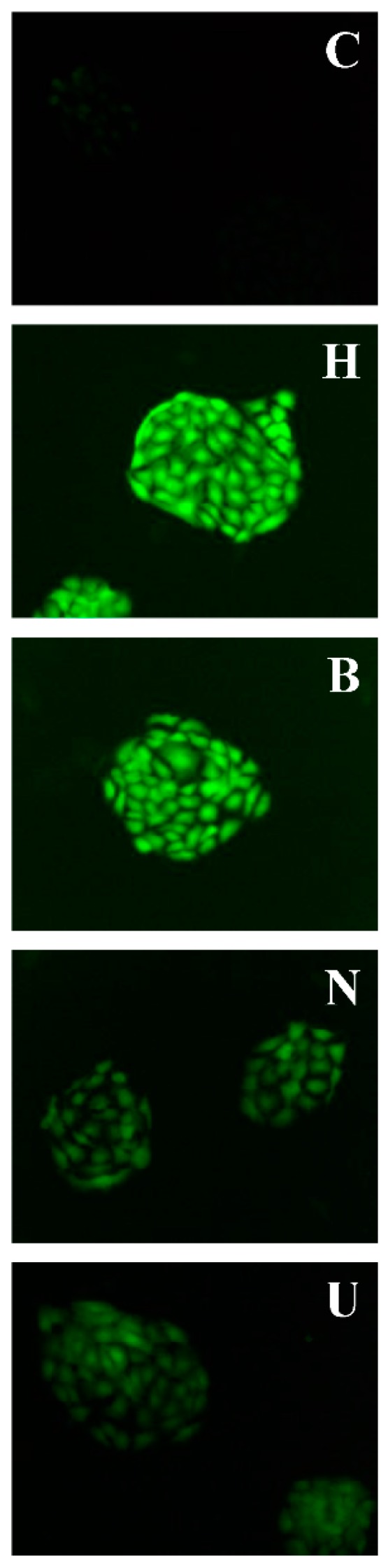
Analysis of reactive oxygen species (ROS) in HaCaT cells. Representative fluorescence photomicrographs (objective 40×) of DCFH-DA staining output via fluorescence microscopy. C: Normal culture group; H: Only oxidative damage group; B: Blank-NLC-treated group; N: UB-NLC-treated group; U: Free UB-treated group.

**Figure 8:**
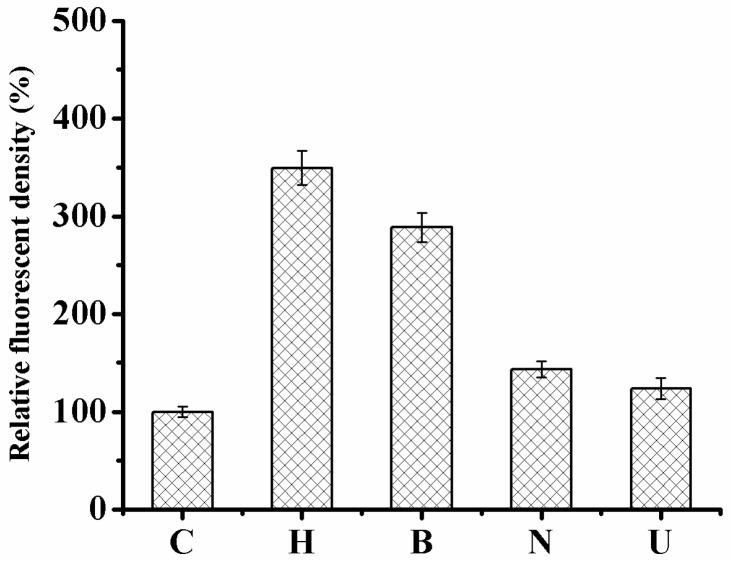
The quantification of fluorescence intensity of the oxidized product DCF in HaCaT cells is presented in the bar graphs. C: Normal culture group; H: Only oxidative damage group; B: Blank-NLC-treated group; N: UB-NLC-treated group; U: Free UB-treated group.

The general nonspecific ROS probe DCFH-DA to measure ROS in HaCaT cells was subjected to samples because of its relative ease of use and stabilization. Control HaCaT cells under normal culture conditions indicated faintly visible signals of ROS (Figure 7 and Figure 8). In comparison to cells incubated only with hydrogen peroxide, ROS generation declined when treated with UB-NLC followed by hydrogen peroxide but revealed no significant differences compared with cells of control. However, cells incubated with Blank-NLC, followed by hydrogen peroxide, showed a significant difference in comparison to normal culture HaCaT cells. Treatment of HaCaT cells with hydrogen peroxide significantly augmented intracellular ROS production in comparison to the untreated control cells. Pre-treatment of cells with UB-NLC diminished the degree of intracellular ROS. It can be concluded that the hydrogen peroxide treatment of HaCaT cells induced oxidative injury, whereas UB-NLC pre-treatment reduced ROS generation.

### 2.5. Effect of UB-NLC on the Activity of Superoxide Dismutase (SOD) and Glutathione Peroxidase (GSH-PX)

SOD is a key antioxidant enzyme that plays a crucial role in the protection of the cells from oxidative stress [1,3]. On the first line of defense against oxidative stress [9], SOD consecutively catalyzes the dismutation of superoxide ion into the less reactive metabolite hydrogen peroxide, which attenuates free radical impairment in the eukaryotic cells [1,3,18,20]. Glutathione peroxidase (GSH-PX) is an enzyme that can protect the cells from oxidative injury [3]. Glutathione (GSH) is a tripeptide and a fundamental provider in cellular antioxidative defense, which serves for scavenging free radicals [20]. The GSH-PX converts hydrogen peroxide into water and oxygen; it also oxidizes glutathione (GSH) to oxidized glutathione (GSSG) [5,13,37], averting the production of reactive hydroxyl radical and protecting the cells against ROS [18].

**Figure 9:**
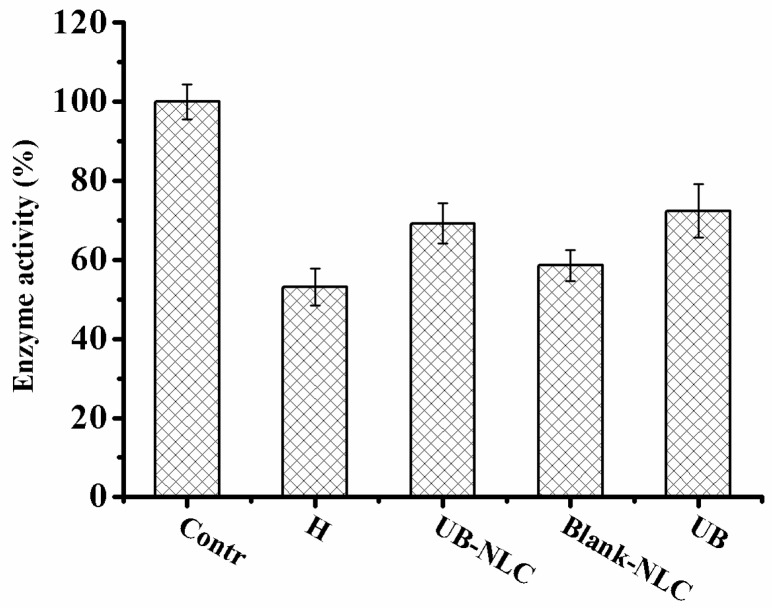
SOD activity of HaCaT cells during culture period with or without UB-NLC.

**Figure 10:**
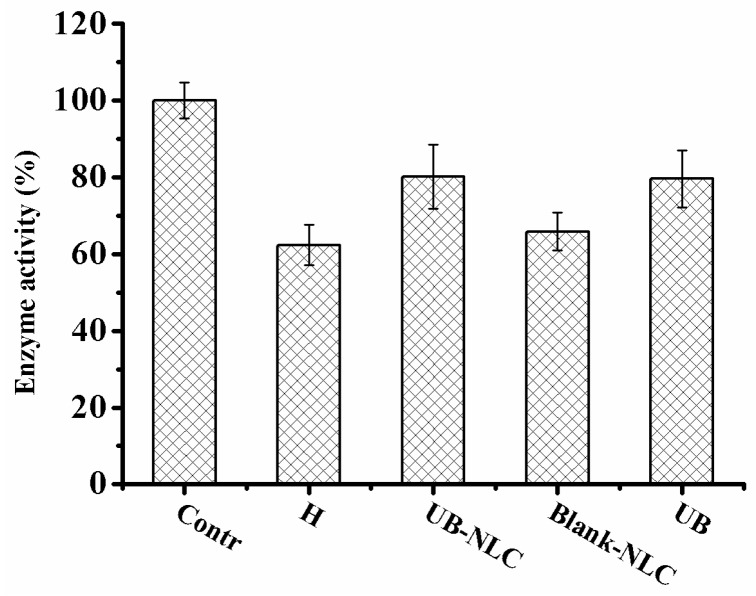
GSH-PX activity of HaCaT cells during culture period with or without UB-NLC.

Hydrogen peroxide treatment reduces the activity of SOD and GSH-PX in HaCaT cells, leading to a reduction in free radical controlling influence, which brings about cell impairment by enhanced superoxide levels. When activity of SOD and GSH-PX of formulation-incubated groups were compared with the hydrogen peroxide-treated group, there was no significant difference in the activity of SOD and GSH-PX between hydrogen peroxide-treated group and Blank-NLC-treated group. The effect of SOD and GSH-PX in the HaCaT cells with UB-NLC pretreatment was significantly higher than in the groups with the Blank-NLC pretreatment. Moreover, a significant enhancement in activity of SOD and GSH-PX was expressed within the UB-NLC-treated group compared with the hydrogen peroxide-incubated group, suggesting the restoration of activity of SOD (Figure 9) and GSH-PX (Figure 10). No significant difference in the effectiveness of SOD and GSH-PX was monitored between the UB-NLC-preincubated group and the UB-pretreated group. UB is a labile compound, and the stability of UB in the NLC-based formation (UB-NLC) obviously improved in comparison to free UB, which has been reported in our previous work [38]. Results suggest that UB-NLC may decrease oxidative stress by ameliorating the scavenging activity of SOD and GSH-PX in HaCaT cells.

### 2.6. Effect of UB-NLC on Lipid Peroxidation

Malondialdehyde (MDA) is a highly reactive three-carbon dialdehyde, generated as a decomposition product of polyunsaturated fatty acids [6,39]. MDA is frequently investigated index of ROS production, which presents a marker of the overall lipid peroxidation degree [9,14,39]. Lipid peroxidation was determined in relation to the quantity of MDA in an admitted assay for measuring the degree of oxidative injury and oxidative stress at the cellular level [13].

**Figure 11:**
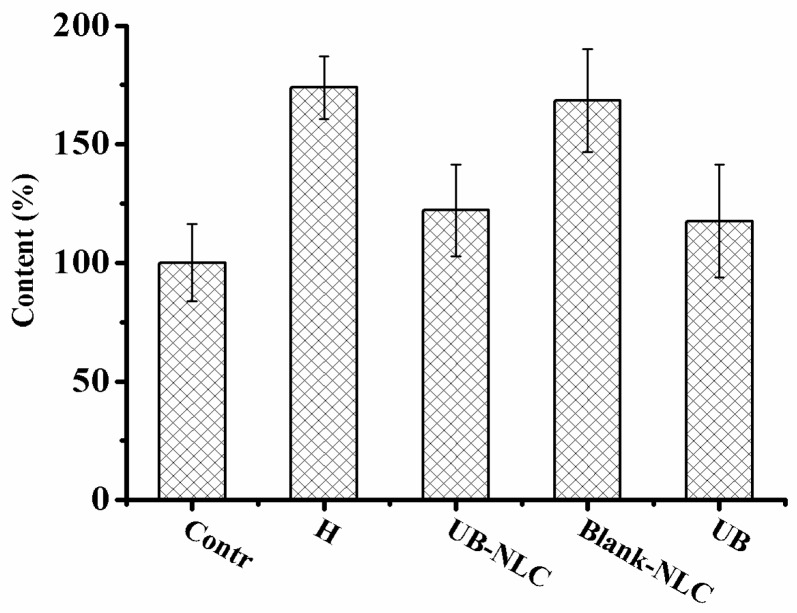
MDA level of HaCaT cells during culture period with or without UB-NLC.

In the current investigation, HaCaT cells treated with hydrogen peroxide experienced peroxidation, leading to augmented production of MDA, which was improved in the company of the UB-NLC pretreatment (Figure 11). It can be concluded that the protective activity of UB-NLC was against the hydrogen peroxide-induced lipid peroxidation in HaCaT cells. Enhanced MDA levels in both the hydrogen peroxide-treated group and Blank-NLC-treated group were detected in the course of the culture process, showing excessive formation of oxidative stress.

UB-NLC augmented the SOD and GSH-PX activity in HaCaT cells during the culture period (Figure 9 and Figure 10). The attenuated SOD and GSH-PX activities in HaCaT cells without UB-NLC pretreatment were accompanied with a significant enhancement in lipid peroxidation (MDA). The data of the current investigation revealed that adding UB-NLC to the culture media diminished lipid peroxidation. In the present study, UB-NLC attenuated the MDA levels, indicating inhibitory activity on lipid peroxidation and production of ROS, paralleled with enhanced GSH and SOD activities, which demonstrated significant improvement of antioxidant defenses.

## 3. Materials and Methods

### 3.1. Materials

UB was obtained from Haotian Bioengineering Technology Co., Ltd. (Xi’an, China). O/100G (Trade name) and O/020G (Trade name) were purchased from Xianglin Enterprise Co., Ltd. (Guangzhou, China). Glycerin monostearate, Octyl and decyl glycerate were provided by Zhengtong Chemical Co., Ltd. (Zhengzhou, Henan, China). Glyceryl triacetate was obtained from Beijing Chemical Reagent Co., Ltd. (Beijing, China). Glyceride and span 20 were supplied by Sinopharm Chemical Reagent Co., Ltd. (Shanghai, China). HPLC grade ethanol was purchased from Fisher Scientific (Fair Lawn, NJ, USA) and used as received. Methanol of HPLC grade at the highest purity available was provided by Merck (Darmstadt, Germany). HaCaT cells were obtained from China Center for Type Culture Collection (CCTCC). Phosphate-buffered saline (PBS), Culture media, and trypsin were obtained from Hyclone (Logan, UT, USA). Fetal bovine serum (FBS) and penicillin-streptomycin were supplied by Gibco (Carlsbad, CA, USA). Ultra-pure water used in the measurements was obtained from a MilliQ-Plus purification system (Schwalbach, Germany).

### 3.2. Preparation of UB-NLC

UB-NLC was prepared via stirring followed by homogenization. The lipid mixture was melted at 65 °C, then added to UB in the lipid phase. Subsequently, the oil phase (the mixture of lipids) was blended with the water phase (aqueous surfactant solution) under stirring. Afterwards, the two-phase system was high-speed stirred using an Ultra-Turrax FM200 (Fluko, Essen, Germany) and subsequently homogenized via a high pressure homogenizer (ATS 100D, ATS Engineering Inc., Ottawa, ON, Canada), and then cooled to ambient temperature.

### 3.3. Size Analysis

NLC size assessment was carried out by Photon Correlation Spectroscopy (PCS) using a Malvern Zetasizer ZS90 (Malvern Instruments, Worcestershire, UK). Experiments were done at 25 °C and at a scattering angle of 90°. The average value of measurements, with a minimum of 12 runs for each sample, was expressed as the final result.

### 3.4. Freeze-Fracture Transmission Electron Microscopy (FF-TEM) Observations

Freeze-fracture transmission electron microscopy (FF-TEM) was employed to characterize the morphology of UB-NLC [40,41,42,43,44]. For freeze-fracture (FF) measurements, droplets of the sample were dropped onto a specimen holder and frozen via a rapid plunge into liquid nitrogen. Then, samples were fractured and replicated using a freeze-etching apparatus (Balzers BAF 400D, Balzers, Liechtenstein). Platinum-carbon was deposited to shadow the replicas. Finally, the prepared samples were loaded onto copper grids and then observed by a transmission electron microscopy (JEOL JEM-2010HR, Tokyo, Japan).

### 3.5. High Performance Liquid Chromatography (HPLC) Analysis

The high performance liquid chromatography (HPLC) analytical technique was used for analysis of UB. The determination was executed by high performance liquid chromatography (Perkin-Elmer, LC-200U, Waltham, MA, USA), coupled with an auto sampler injector. The mobile phase comprised of a system maintained at 10% of methanol and 90% of ethanol set at a flow rate of 1.5 mL/min. The quantity of UB present in the samples was measured at a wavelength of 275 nm. The mobile phase was filtered through a filter membrane prior to use.

### 3.6. Skin Deposition Study

Skin deposition was investigated with a Franz diffusion cell using rabbit skin. Japanese white rabbits were ethically sacrificed by cervical dislocation and the full thickness skin was removed and entirely depilated [31]. The dermal surface was thoroughly eliminated of subcutaneous tissues without impairing the epidermal surface [28]. Rabbits skin was placed between donor and receiver compartments of the Franz diffusion cells, with the stratum corneum in contact with the donor compartment and the dermis side facing toward the receptor medium. The UB-NLC was positioned in the donor compartment [31]. The system was kept at 32 ± 1 °C to imitate skin temperature during measurement by means of circulation of a thermo-regulated outer water jacket surrounding the diffusion cells; simultaneously, the diffusion solution was stirred constantly using a magnetic stirrer [31]. At selected time intervals, samples were removed and instantly substituted by an equal volume of fresh buffer medium to keep both steady volume and sink conditions [28,45,46]. After achievement of skin deposition research, the skin mounted between donor and receiver compartments was cautiously taken off [31]. The collected skin sections were cut into pieces and sonicated in ethanol. Then, the skin homogenate was filtered through a filter membrane [28]. The obtained samples to be determined for amounts of UB were analyzed by HPLC methods [27].

### 3.7. Cell Culture

HaCaT cells, immortalized human keratinocytes, were cultured in Minimum Essential Medium (MEM) containing 10% fetal bovine serum (FBS). Cells were maintained in the presence of 5% CO_2_ atmosphere at 37 °C.

### 3.8. Cell Viability Assay

The effects of UB-NLC exposure on cell viability were estimated using the 3-(4,5-dimethylthiazol-2-yl)-2,5-diphenyltetrazolium bromide (MTT) assay. Cells were seeded into a 96-well plate and incubated for 24 h. Cells were pretreated with different samples for selected time intervals and then cells were exposed to oxidative stress caused by ROS (hydrogen peroxide). Subsequently, MTT was added into each well and incubated for another 4 h. Then, after abandoning the supernatant, DMSO was added and the absorbance of each well was assessed at the wavelength of 492 nm via an automated microplate reader (Thermo Fisher Scientific Co., Ltd., Waltham, MA, USA). Furthermore, the cell viability was expressed as a percentage relative to the control.

### 3.9. Time-Lapse Imaging Assays

Cells were exposed to a time-lapse recording under Time Lapse Imaging System (Nikon, Tokyo, Japan) enclosed in a cell culture incubator; 5% CO_2_ atmosphere and 37 °C temperature were adopted as culture conditions. Image acquisition was carried out at diverse focal positions for each cell culture plate. Micrographs were obtained in the phase-contrast mode [47,48].

### 3.10. Measurement of Reactive Oxygen Species (ROS)

Cells were seeded in culture plates for 24 h in a CO_2_ incubator. The supernatant was discarded at the end of treatment and cells were washed with PBS buffer and incubated with DCFH-DA at 37 °C in the dark. DCF fluorescence intensity was obtained with a fluorescence microscope (Nikon Corporation, Tokyo, Japan) [10,32,49,50].

### 3.11. Assay of Superoxide Dismutase (SOD) Activity

SOD in the sample diminished the complete superoxide anion concentration, thus reducing the colorimetric signal and absorbance at 550 nm by UV-visible double beam spectrophotometer (UV-2450, Shimadzu, Kyoto, Japan). The activity of SOD was determined using a superoxide dismutase commercial assay kit (Nanjing Jiancheng Bioengineering Institute, Nanjing, China), according to the manufacturer’s instructions.

### 3.12. Glutathione Peroxidase (GSH-PX) Activity Assay

Glutathione peroxidase (GSH-PX) activity was based upon the reaction of reduced glutathione (GSH) transformation to oxidized glutathione (GSSG). GSH-PX activity was spectrophotometrically determined at 412 nm on a UV-visible double beam spectrophotometer (UV-2450, Shimadzu, Japan). The procedures of GSH-PX activity assay were performed using a commercially available kit (Nanjing Jiancheng Bioengineering Institute, Nanjing, China) according to the provided manufacturer’s protocol.

### 3.13. Determination of Malondialdehyde (MDA)

Malondialdehyde (MDA) as the main product of lipid peroxidation (LPO) was assessed using a commercially available colorimetric assay MDA detection kit (Nanjing Jiancheng Bioengineering Institute, Nanjing, China). The absorbance was determined at 532 nm by an UV-visible double beam spectrophotometer (UV-2450, Shimadzu, Japan). All measurements were conducted according to the manufacturer’s instructions.

### 3.14. Statistical Analysis

All experiments were carried out in triplicate unless otherwise indicated. Statistical difference of data was determined via Student’s *t*-test with a probability value of less than 0.05.

## 4. Conclusions

In this investigation, ubidecarenone was successfully encapsulated into a nanostructured lipid carrier by high pressure homogenization, preparing a stable and topically attractive aqueous formation (UB-NLC). The attained results highlight that NLC is a good nanocarrier for ubidecarenone. Percutaneous penetration investigation indicated that ubidecarenone-loaded NLC can retain a significantly higher quantity of drug in the skin. Ubidecarenone retained its antioxidant activity after its incorporation into a nanostructured lipid carrier. The inherent ROS-scavenging effect and inducement of the antioxidant enzymes activities exposed to UB-NLC were responsible for protection against hydrogen peroxide-mediated oxidative impairment on HaCaT cells. Administration of UB-NLC attenuated oxidative stress via ameliorating activity of vital antioxidant enzymes (for example, SOD and GSH-PX) in an experimental model. Therefore, UB-NLC is an appealing antioxidant formulation with significant preventive and/or therapeutic effects in the improvement of oxidative damage via the regulation of oxidative indications (such as MDA) and the enhancement of activity of antioxidant enzymes. On the basis of the findings of the present investigation, it can be concluded that ubidecarenone-loaded NLC is an attractive nanocarrier for topical delivery. These results indicate that UB-NLC could be a potential candidate for the treatment of oxidative stress-related disorders.
